# Predictors of Change in the Anemia Status Among Pulmonary Tuberculosis Patients Following Anti-tuberculosis Treatment in Puducherry, India

**DOI:** 10.7759/cureus.44821

**Published:** 2023-09-07

**Authors:** Jovita Leon, Sonali Sarkar, Debdatta Basu, Nivedita Nanda, Noyal M Joseph

**Affiliations:** 1 Preventive and Social Medicine, Jawaharlal Institute of Postgraduate Medical Education & Research, Puducherry, IND; 2 Pathology, Jawaharlal Institute of Postgraduate Medical Education & Research, Puducherry, IND; 3 Biochemistry, Jawaharlal Institute of Postgraduate Medical Education & Research, Puducherry, IND; 4 Microbiology, Jawaharlal Institute of Postgraduate Medical Education & Research, Puducherry, IND

**Keywords:** anemia, nutritional status, pulmonary tuberculosis, hemoglobin, dietary intake, anti-tubercular treatment

## Abstract

Background

Pulmonary tuberculosis (PTB) is commonly associated with reversible peripheral blood abnormalities. The evolution of tuberculosis (TB)-associated anemia with anti-tuberculosis treatment (ATT) has not been well elucidated. This study aimed to compare the hematological profiles at the start and end of the ATT among new sputum smear-positive (NSP) PTB patients in Puducherry, India.

Methods

A prospective cohort study was conducted in the 10 urban primary health centers of Puducherry from 2017 to 2020. All the NSP PTB participants aged ≥18 years registered under the National Tuberculosis Elimination Program (NTEP) were contacted within two weeks of the start of the ATT. All eligible participants were enrolled, and they were followed up till the end of ATT (180 days). Hematological profiles and anthropometric measurements were compared at the start and end of the ATT. Binomial logistic regression analysis was used to assess the predictors of changes in the anemia status at the start and end of the ATT.

Results

Out of 176 NSP PTB participants, 145 were followed up after treatment. Initially, 63% (111/176) patients had anemia, which decreased to 44% (64/145) by the end of treatment. The risk factors for a negative change in hemoglobin levels were female gender, below poverty level, underweight, and reduced iron intake. The adjusted risk ratios (ARRs) were 1.53 (1.24-1.88), 1.18 (1.01-1.38), 1.29 (1.02-1.64), and 1.26 (1.05-1.51),respectively.

Conclusion

ATT may lead to the resolution of TB-associated anemia. Moreover, female gender, possession of a red ration card, being underweight, and reduced iron intake were identified as risk factors for negative changes in hemoglobin levels during treatment.

## Introduction

Prior to the onset of the coronavirus disease 2019 (COVID-19) pandemic, tuberculosis (TB) was the most common cause of mortality resulting from a single infectious agent. Notably, both TB and COVID-19 manifest as respiratory tract infections [1]. COVID-19 caused a spike in TB cases and deaths worldwide [1]. The Centers for Disease Control and Prevention (CDC) reported that TB incidence in 2020 was 20% lower than in 2019, possibly due to reduced transmission and undetected cases [2]. India was one of the eight countries responsible for over two-thirds of global new TB cases in 2021, despite COVID-19 [1]. Though *Mycobacterium tuberculosis* (MTB) can affect many organs, it primarily causes disease in the lungs causing pulmonary tuberculosis (PTB). Lungs are the most commonly affected organs, and patients with PTB are studied because the effect of treatment is easily ascertained through sputum conversion.

Anemia is one of the common risk factors and hematological abnormalities associated with TB [3]. In 2019, Barzegari et al., conducted a meta-analysis study to determine the prevalence of anemia among TB patients, which was estimated to be 61.53% [4]. TB infection, by inducing systemic inflammation, impairs iron homeostasis and leads to anemia [5]. The reduction of erythropoiesis by inflammatory indicators, malabsorption syndrome, and dietary deficiencies are elucidated as the underlying pathophysiology of anemia in TB patients [6,7].

Anemia can significantly impact TB in several ways. Several studies have shown that anemia is associated with worse TB disease outcomes, such as increased mortality rates and longer treatment times [8-10]. Anemia can also lead to other complications, such as lung injury due to enlargement of infectious zones in lungs, which can further worsen TB disease outcomes [11]. In addition, anemia can make the treatment of TB more difficult for patients by increasing the side effects of TB medications such as gastrointestinal distress and hepatotoxicity [12]. Therefore, early diagnosis and treatment of anemia in patients with TB are crucial for improving TB disease outcomes.

Anti-TB treatment (ATT) is also known to reduce anemia in TB patients [13]. Understanding the alterations in haematological parameters due to antitubercular therapy is critical for decisions on whether or not to treat anemia in TB patients for improving TB treatment outcomes and reducing mortality. Even though anemia has been extensively studied in the context of TB, only a few studies in India have explored changes in haematological parameters during ATT. However, these studies report observation until the start of intensive phase (IP) or the end of IP and not after the completion of ATT [7,14,15]. Some studies have examined the prevalence and evolution of anemia associated with anti-tubercular treatment as well as the impact of persistent anemia on systemic recovery [16,17]. Other sources provide information on different types of anemia, such as iron deficiency anemia and anemia of chronic disease at the initiation of anti-tubercular treatment, but there is limited insight into factors, especially nutritional intake, associated with haematological changes during the course of ATT [18,19]. Hence, we undertook this study to describe the changes in haematological parameters and their predictors over the course of ATT among new sputum smear-positive (NSP) TB patients in Puducherry.

## Materials and methods

Study design and setting

Puducherry consists of 15 urban primary health centres (UPHCs) and 24 rural primary health centres (RPHCs) [20]. The study was conducted between 2017 to 2020 in 10 UPHCs located within 15 km of the parent institute Jawaharlal Institute of Postgraduate Medical Education & Research in Puducherry, India. According to Census 2011, the total population of Puducherry was 1,248,000. TB diagnosis, treatment, and control services such as monitoring of treatment outcomes and screening of household contacts were provided free of charge through the National Tuberculosis Elimination Program (NTEP). Under this program, 7 TB units (TUs) and 27 designated microscopy centres (DMCs) were functional in Puducherry. After screening for comorbidities, patients diagnosed with TB at the DMCs are referred to the nearby PHCs for initiation of treatment. As part of a daily regimen, patients received antitubercular drugs based on their body weight [21]. Patients with anemia were not provided with iron supplements. Considering undernutrition as one of the risk factors for TB, in April 2018, the Ministry of Health and Family Welfare, Government of India, announced the scheme “Nikshay Poshan Yojana” for nutritional support to TB patients, who receive an incentive of Rs. 500 per month as direct benefit transfer (DBT) [22].

Study population

The study included participants aged 18 years or older, registered under the NTEP in selected 10 PHCs within two weeks of initiation of treatment. Pregnant or lactating women, HIV-positive individuals, physically challenged individuals, those who had undergone blood transfusion, and those with other chronic illnesses were excluded from the study.

Sample size and sampling technique

Participants in this study were a part of a cohort aimed at characterizing types of anemia among TB patients at treatment initiation and completion. The sample size of 176 was calculated using OpenEpi, version 3.01, based on a 75.9% prevalence of anemia of inflammation at the beginning of anti-tuberculosis treatment, a 0.05 α-error, 7% absolute precision, and a 20% non-response rate [23].

Study procedure

The list of eligible participants was obtained from the NTEP registers maintained in selected PHCs from August 2017 to October 2019 and followed until March 2020. Participants were contacted at their convenience either at the PHC or their home. Recruitment began after obtaining informed consent. A pre-tested questionnaire was used to collect information on various socio-demographic details, including age, sex, residence, marital status, and occupation. Risk behaviours such as alcohol and smoking were assessed using the Alcohol Use Disorders Identification Test for Consumption (AUDIT-C) scale and Fagerström Test for Nicotine Dependence (FTND), along with 24-hour dietary recall and nutritional status measurements, including body mass index (BMI) and mid-upper arm circumference (MUAC). Study participants were assessed at two time points: (i) at the start of the ATT (start of the intensive phase) and (ii) at the end of the ATT within two weeks following 180th day. ATT was initiated according to the National Tuberculosis Elimination Program. At each visit, 2 mL of venous blood was collected from study participants using an ethylenediamine tetraacetic acid (EDTA) tube to conduct complete blood count (CBC) testing. The estimation of hemoglobin (Hb), red blood cell (RBC), hematocrit (HCT), mean corpuscular volume (MCV), mean corpuscular hemoglobin (MCH), mean corpuscular hemoglobin concentration (MCHC), red cell distribution width (RDW), neutrophils (N), basophils (B), lymphocytes (L), monocytes (M), platelets (P) and peripheral smear was done at the start and end of ATT and analyzed via autoanalyzer XTi 4000i (Sysmex Corporation, Kobe, Japan) at the Department of Pathology. BMI was recorded at the start and end of the ATT to determine the nutritional status using a standard weighing machine. Dietary assessment was done using the 24-hour recall method at the start and end of ATT. The participants were asked to recall all the food they had taken the previous day since the time they woke up. Data on dietary intake was analyzed using DietSoft, version 1.2.0 (Department of Dietetics, AIIMS, and Invincible IDEAS Co., India), a software package to determine the content of macronutrients and micronutrients in the diet. The results were compared with the recommended dietary allowance (RDA) 2010 for Indians by the National Institute of Nutrition (Indian Council of Medical Research), Hyderabad, to assess the adequacy of dietary intake.

Operational definitions

Alcohol Use

The consumption of any form of alcohol (one standard drink) in the past year was assessed using AUDIT-C [24].

Tobacco Use

The use of smoke or smokeless form of tobacco in the past year was assessed using FTND [25].

Anemia

According to the WHO classification, the study participants were classified as anemic when the hemoglobin levels were less than 13 g/dL and 12/dL, for males and females, respectively [26].

Mean Corpuscular Volume

Normal mean corpuscular volume (MCV) values range from 80 to 100 fL. Values below 80 fL are considered decreased, while values above 100 fL are considered increased [27].

Normocytic Normochromic Anemia

Normocytic normochromic anemia (NCNC) is characterized by an MCV within the normal range of 80-100 fL.

Microcytic Hypochromic Anemia

Microcytic hypochromic anemia (MCHC) is identified by an MCV below 80 fL.

Macrocytic Anemia

Macrocytic anemia (MG) is diagnosed when the MCV level is above 100 fL.

Start of the ATT

The start of the ATT involved study participants who were enrolled within two weeks of initiating ATT.

End of the ATT

The end of the ATT refers to the follow-up of study participants within two weeks of completing the 180-day course of ATT.

Positive Hb Status

Positive Hb status pertains to participants whose hemoglobin levels improved from the anemic to non-anemic status during the course of the treatment, or those who remained non-anemic from the start to the end of ATT.

Negative Hb Status

Negative Hb status refers to participants whose Hb status remained anemic from the beginning to the end of the ATT, as well as those who developed anemia by the end of the ATT after starting with a non-anemic status.

Dietary Deficiency

Dietary deficiency is defined as nutritional intakes falling below the recommended dietary allowances for specific nutrients. Energy requirements and RDAs for males and females for various nutrients are as follows: energy (male), 2320 kcal; energy (female), 1900 kcal; protein (male), 60 g/day; protein (female), 55 g/day; iron (male), 17 mg; iron (female), 21 mg; calcium, 600 g; vitamin C, 40 mg; vitamin B12, 1.0 µg; folic acid, 100 µg (RDAs for calcium, vitamin C, vitamin B12, and folic acid are the same for both genders) [28].

Mid-Upper Arm Circumference

Mid-upper arm circumference (MUAC) values below 23 cm for males and 22 cm for females are considered low [29].

Statistical analysis

The data analysis was performed using Stata, version 14 (StataCorp LP, College Station, TX) and sankey diagram using RStudio, version 4.1.3 (Posit, Boston). Categorical variables were described using frequency (n) and percentage (%). Continuous variables were described using the median and interquartile range (IQR). Wilcoxon's signed-rank test was used to compare the hematological parameter and nutrient intake at the start and end of ATT. Variables with a p-value <0.2 in bivariate analysis were entered into the multivariable logistic regression model. We performed log binomial regression analysis to determine factors independently associated with a negative Hb status. The results are presented in the form of adjusted relative risk ratios and 95% confidence intervals (CIs). A p-value <0.05 was considered to be statistically significant.

## Results

During the study period, a total of 190 NSP PTB patients were registered under NTEP; 176 of them were eligible for the study. The reasons for exclusion were non-willingness to participate in the study (n=5), previous ATT for pulmonary tuberculosis (n=6), and inappropriate address (n=3). Among the 176 participants who were enrolled in the study, 18% (n=31) were lost to follow-up (LTFU) in the follow-up visits. LTFU was higher among males compared to females (25% vs. 8%) and the reasons included death (n=2), non-consent (n=22), and default (n=7).

Characteristics of the study participants

Of the 176 participants enrolled in the study, 73.2% were males; a higher proportion (71.0%) belonged to the age group of 30-60 years. About 14% of the participants did not have any formal education, 55.1% were employed and 64.2% were from families below poverty line.

Clinical and lifestyle risk factors among the study participants

At baseline, alcoholism, smoking and diabetes mellitus were reported by 44.9%, 38.6%, and 46% of the participants, respectively. However, by the end of ATT almost all the participants who were consuming alcohol were converted to the low-risk (score: 0-7) category and about 10% continued smoking. Undernourishment was found in 44.9% of the participants at baseline, 36% of whom improved by the end of ATT (Table 1).

**Table 1:** Distribution of anemia based on socio-demographic characteristic of new sputum smear-positive pulmonary tuberculosis patients at the start and end of the ATT ATT: anti-tuberculosis treatment; APL: above poverty line; BPL: below poverty line; DM: diabetes mellitus; BMI: body mass index; MUAC: mid-upper arm circumference; AUDIT-C scale: Alcohol Use Disorders Identification Test for Consumption scale AUDIT-C scale: lower risk (0-7); increasing risk (8-15); high risk (16-19); possible dependence (20+) Fagerström Test for Nicotine Dependence: very low (0-2); low (3-4); medium (5); high (6-7); very high (8-10) MUAC: male, <23 cm; female, <22 cm, considered as low *Separated/divorced/widow/widower

Variables	Start of the ATT, n=111	End of the ATT, n=64
	Anemia, n (%)	Anemia, n (%)
Gender		
Male	72 (55.81)	32 (30.77)
Female	39 (82.98)	32 (78.05)
Age (years)		
18-30	26 (72.22)	17 (51.52)
30-60	72 (57.60)	39 (39.39)
>60	13 (86.67)	08 (61.54)
Religion		
Hindu	97 (62.58)	55 (43.65)
Christian	08 (57.14)	06 (46.15)
Muslim	06 (85.71)	03 (50.00)
Education		
No formal education	18 (72.00)	07 (43.75)
Primary, middle, and secondary stage	56 (64.37)	28 (39.44)
Senior secondary education, bachelor's, master's or equivalent	37 (57.81)	29 (50.00)
Occupation		
Employed	50 (51.55)	24 (30.77)
Unemployed	61 (77.22)	40 (59.70)
Type of ration card		
Yellow	32 (50.79)	18 (32.73)
Red	79 (69.91)	46 (51.11)
Marital status		
Married	78 (61.90)	46 (44.66)
Unmarried	26 (68.42)	15 (44.12)
Others*	7 (58.33)	03 (37.50)
AUDIT-C score		
Lower risk	70 (65.42)	62 (43.46)
Increasing risk	27 (62.79)	0
Higher risk	6 (75)	0
Possible dependence	8 (44.44)	0
Fagerström Test for Nicotine Dependence		
Very low	84 (75.68)	64 (44.76)
Low	13 (11.71)	0
Medium	6 (5.41)	0
High	7 (6.31)	0
Very high	1 (0.90)	0
DM		
Yes	42 (51.85)	24 (36.36)
No	69 (72.63)	40 (50.63)
BMI		
Underweight (<18.5)	67 (84.81)	13 (48.15)
Normal (18.5-22.9)	34 (50.00)	42 (49.41)
Overweight (23-24.9)	04 (30.77)	05 (38.46)
Obesity (≥25)	06 (37.50)	04 (20.00)
MUAC		
Normal	49 (51.58)	42 (40.38)
Deficient	62 (76.54)	22 (53.66)

Changes in morphological types of anemia

**Figure 1:**
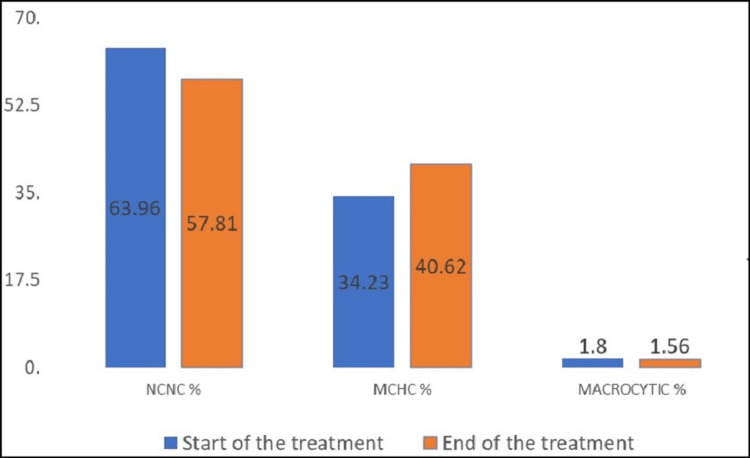
Types of anemia at the start and end of the treatment NCNC anemia: normocytic normochromic anemia; MCHC anemia: microcytic hypochromic anemia

Figure 1 depicts the morphological categories of anemia at the start and end of ATT. About 63.07% (111/176) had anemia, consisting of 86.4% (96/111) with mild anemia, 12.6% (14/111) with moderate anemia, and 0.9% (1/111) with severe anemia. Based on MCV levels, 63.9% (71/111) had normocytic normochromic anemia, 34.23% (38/111) had microcytic hypochromic anemia, and 1.8% (2/111) had macrocytic anemia.

Following treatment, anemia was reduced by 44%, with 84.3% (54/64) in mild cases, 12.5% (8/64) in moderate cases, and 3.15% (2/64) in severe cases. Normocytic normochromic anemia decreased to 57.81% (37/64), while microcytic hypochromic anemia increased from 34.2% to 40.6%. Rare cases of macrocytic anemia were observed, with 1.8% and 1.6% at the start and end of ATT.

Changes in hemoglobin status

The median (IQR) Hb concentration of the study participants at the start of the ATT was 11.9 (10.5-13.4 g/dL), which improved to 13 (11.4-14.6) g/dL at the end of ATT. Though it was statistically significant, the median (IQR) Hb concentration improved minimally among the anemic group (start of ATT, 10.9 (9.8-11.8) g/dL vs. end of ATT, 11.1 (10.15-11.8) g/dL) (Table 2).

**Table 2:** Hematological parameters for new sputum smear-positive pulmonary tuberculosis patients at the start and end of treatment ATT: anti-tuberculosis treatment; Hb: hemoglobin; RBC: red blood cell; HCT: hematocrit; MCV: mean corpuscular volume; MCH: mean corpuscular hemoglobin; MCHC: mean corpuscular hemoglobin concentration; RDW: red cell distribution width The values for hematological parameters are reported in median (IQR).

Hematological parameters	Start of the ATT		End of the ATT	
	Anemia, n=111	No anemia, n=65	Anemia, n=64	No anemia, n=81
Hb (g/dL)	10.9 (9.8-11.8)	13.9 (13.3-14.6)	11.1 (10.15-11.8)	14.5 (13.4-15.2)
RBC (µL)	4.28 (3.86-4.49)	4.98 (4.67-5.36)	4.31 (3.96-4.59)	4.89 (4.55-5.11)
HCT (%)	34 (31.3-36.4)	41.75 (39.3-43.5)	34.9 (32.75-36.5)	42.3 (40.2-44.2)
MCV (fL)	81.6 (75.4-86.1)	82.3 (78.45-88.7)	81.45 (75.85-89.1)	87.5 (83.6-92)
MCH (pg)	26.3 (23.8-28.5)	28.1 (26.25-29.75)	26.75 (23.35-28.8)	29.7 (28.2-31.4)
MCHC (gm%)	31.8 (31.1-32.8)	33.55 (32.6-34.35)	31.9 (30.7-33)	34.05 (33.1-35.1)
RDW (%)	15 (13.7-17.3)	13.6 (12.9-14.9)	15.7 (14.1-18.4)	13.45 (12.9-14.5)
WBC (µL)	9.41 (7.74-10.75)	8.97 (7.67-10.67)	7.06 (5.79-8.35)	6.67 (5.78-7.71)
Neutrophils %	73.35 (66.6-78.6)	68.2 (62.5-74.1)	60.3 (55.2-66.2)	56.45 (48.6-62.3)
Eosinophils %	1.95 (1-4.1)	2.9 (1.8-4.8)	2.85 (1.6-6.15)	4.15 (2.35-7.05)
Basophils %	0.13(0.-0.1)	0.07 (0-0.1)	0 (0-0.2)	0.1 (0-0.2)
Lymphocytes %	15.3 (12.1-20.3)	18.8 (14.7-25.1)	29.2 (20.4-34.5)	28 (24.8-33.9)
Monocytes %	8 (6.1-11)	8.5 (5.8-9.8)	7.8 (6.4-9.9)	7.2 (6-8.7)
Platelets (µL)	414 (327-513)	343 (290-418)	261 (219.5-361)	240 (188-283)

**Figure 2:**
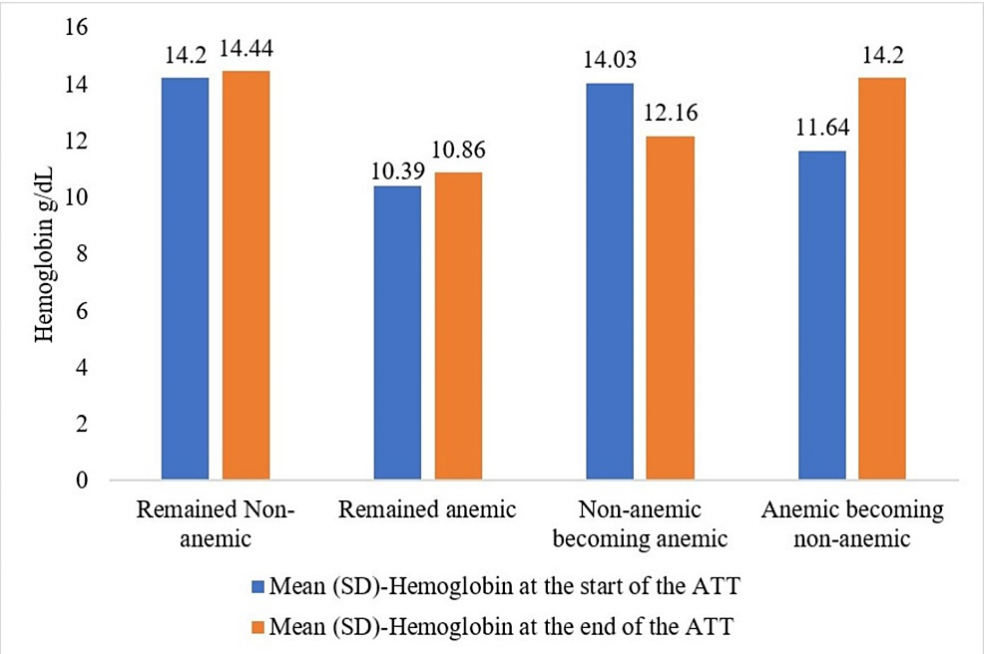
Bar graph showing the average variation in hemoglobin levels among the four groups The 'remained non-anemic' group included participants whose hemoglobin levels remained within the normal range both at the beginning and end of the anti-tuberculosis treatment (ATT). The 'remained anemic' group included participants whose hemoglobin levels stayed below the normal range at both the start and end of the ATT. The 'non-anemic becoming anemic' group included those whose hemoglobin levels decreased from the normal range by the end of the ATT. The 'anemic becoming non-anemic' group included participants whose hemoglobin levels improved toward the normal range during the course of the ATT.

To understand the impact of ATT and its effect on the Hb status, at the start and end of ATT, the study participants were categorized into four groups: (i) remained non-anemic, (ii) remained anemic, (iii) non-anemic becoming anemic, and (iv) anemic becoming non-anemic. Changes in the Hb levels in the four groups are shown in Figure 2.

**Figure 3:**
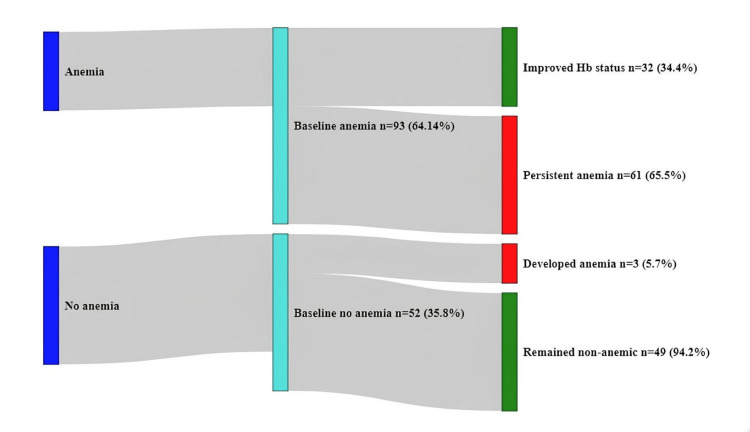
Change in the anemia status observed at the end of the anti-tuberculosis treatment

Of the 111 study participants found to be anemic at the time of diagnosis of TB, 61 (45%) continued to be anemic at the end of ATT. For only 32 (22%) study participants, the Hb status improved. Surprisingly, 3 (2%) study participants who were non-anemic at baseline developed anemia at the end of the ATT (Figure 3).

Change in the nutrient intake at the start and end of the ATT

Initially, the median (IQR) daily energy intake was 1043.1 (IQR 1401.34-1738.71) kcal, almost 1000 kcal below the recommended daily allowance of 2320 kcal. However, by the end of the ATT, the energy intake had increased to a median of 1817.8 kcal (IQR 1988.6-2163.8). Additionally, a third of the participants had reduced protein intake, 46.1 (19.02) g/day, compared to the RDA (60.0 g/day), and micronutrient deficiencies in iron, calcium, vitamin C, vitamin B12, and folic acid were also observed. These deficiencies were found to be lower among participants who were anemic at the start of the ATT (Table 3). Although food consumption improved by the end of the ATT, deficiencies in minerals and protein intake persisted.

**Table 3:** Distribution of anemia based on the nutrient intake of NSP PTB patients at the start and end of treatment ATT: anti-tuberculosis treatment; NSP: new sputum smear-positive; PTB: pulmonary tuberculosis The results were compared with the recommended dietary allowance (RDA) 2010 for Indians by National Institute of Nutrition (Indian Council of Medical Research), Hyderabad, to assess the adequacy of the dietary intake among the anemic and non-anemic NSP PTB patients at the start and end of the ATT. *Median (IQR)

Nutrients	Start of the ATT		End of the ATT	
	Anemia (n=111)	No anemia (n=65)	Anemia (n=64)	No anemia (n=81)
Energy (k/cal)	1421.78 (1052.7-1753.99)*	1345.90 (1025.24-1753.99)	1918.8 (1758.71-2063.48)	2085.52 (1926.14-2245.74)
Protein (g/day)	45.93 (17.68)	46.25 (21.26)	62.42 (11.79)	65.41 (7.66)
Iron (mg)	10.94 (4.29)	11.60 (4.60)	15.69 (4.05)	16.70 (3.29)
Calcium (g)	338.68 (137.99)	353.31 (138.83)	374.32 (145.68)	445.64 (140.84)
Vitamin C (mg)	27.41 (14.21-40.13)*	28.84 (15.56-39.42)	34.23 (22.60-45.20)	38.09 (28.32-45.42)
Vitamin B12 (µg)	0.07 (0-0.14)*	0.07 (0-0.15)	0 (0-0.14)	0.06 (0-0.21)
Folic acid (µg)	3.09 (0.42-5.76)*	2.88 (0-5.76)	0.49 (0-8.12)	3.17 (0-8.9)

Risk factors associated with the negative hemoglobin status at the end of the ATT

Anemia was persistent in 61 of the study participants; three participants developed anemia. A binary logistic regression was performed to predict the factors associated with persistent anemia and those who developed anemia. Female gender (ARR = 1.53, 95% CI: 1.24-1.88), undernutrition (ARR = 1.29, 95% CI: 1.02-1.64), red ration card (APR = 1.18, 95% CI: 1.01-1.38), and low iron intake (APR = 1.26, 95% CI: 1.05-1.51) were significantly associated with a negative change in the Hb status (Table 4).

**Table 4:** Factors of socio-demographic status linked to adverse changes in hemoglobin levels among new sputum smear-positive pulmonary tuberculosis patients at the end of treatment Hb: hemoglobin; URR: unadjusted risk ratio; ARR: adjusted risk ratio; AUDIT-C scale: Alcohol Use Disorders Identification Test for Consumption scale; DM: diabetes mellitus; BMI: body mass index; MUAC: mid-upper arm circumference AUDIT-C scale: low risk (0-7); increasing risk (8-15); high risk (16-19); possible dependence (20+) Fagerström Test for Nicotine Dependence: very low (0-2); low (3-4); medium (5); high (6-7); very high (8-10) MUAC: male, <23 cm; female, <22 cm, considered as low

Variables	Total		Change in the Hb status				URR (95% CI)		p-value		ARR (95% CI)		p-value	
			Negative Hb status		Positive Hb status									
			(n=64)		(n=81)									
Age (years)														
18-30	36		17 (51.52)		16 (48.48)		1.30 (0.86-1.97)		0.2		0.84 (0.69-1.02)		0.08	
31-60	125		39 (39.39)		60 (60.1)		1							
>60	15		08 (61.54)		05 (38.46)		1.56 (0.95-2.56)		0.07		1.13 (0.83-1.52)		0.42	
Gender														
Male	129		32 (30.77)		72 (69.23)		1							
Female	47		32 (78.05)		09 (21.95)		2.53 (1.82-3.53)		<0.001		1.53 (1.24-1.88)		<0.001	
Religion														
Hindu	126		55 (43.65)		71 (56.35)		1							
Christian	13		06 (46.15)		07 (53.85)		1.05 (0.56-1.96)		0.86					
Muslim	06		03 (50)		03 (50)		1.14 (0.50-2.61)		0.74					
Education														
No formal education	16		07 (43.75)		09 (56.25)		1.10 (0.59-2.07)		0.74					
Primary, middle stage and secondary	71		28 (39.44)		43 (60.56)		1							
Senior secondary education, bachelor's or master's or equivalent	58		29 (50.00)		29 (50.00)		1.26 (0.86-1.86)		0.22					
Occupation														
Employed	78		24 (30.77)		54 (69.23)		1							
Unemployed	67		40 (59.70)		27 (40.30)		1.94 (1.31-2.85)		<0.001		1.14 (0.98-1.34)		0.08	
Type of ration card														
Red	90		46 (51.11)		44 (48.89)		1.56 (1.01-2.39)		0.04		1.18 (1.01-1.38)		0.03	
Yellow	55		18 (32.73)		37 (67.27)		1							
Marital status														
Married	103		46 (44.66)		57 (55.34)		1.19 (0.47-2.98)		0.71					
Unmarried	34		15 (44.12)		19 (55.88)		1.17 (0.44-3.10)		0.74					
Others*	08		03 (37.50)		05 (62.50)		1							
AUDIT-C scale														
Lower risk		93		45 (48.39)		48 (51.61)		1.805 (0.48-6.71)		0.378		1.22 (0.91-1.64)		0.79
Increasing risk		36		13 (36.11)		23 (63.89)		3.33 (0.85-13.02)		0.08		1.38 (0.88-2.17)		0.15
Higher risk		06		04 (66.67)		02 (33.33)		2.41 (0.68-8.50)		0.16		1.13 (0.85-1.51)		0.37
Possible dependence		10		02 (20.00)		08 (80.00)		1						
Fagerström Test for Nicotine Dependence														
Very low		120		53 (44.17)		67 (55.83)		1.35 (0.64-2.85)		0.419				
Lower risk		12		05 (41.67)		07 (58.33)		0.94 (0.46-1.89)		0.87				
Medium		05		03 (60.00)		02 (40.00)		0.75 (0.23-2.38)		0.631				
Higher risk		06		02 (33.33)		04 (66.67)		1						
Very high		02		01 (50.00)		01 (50.00)		1.13 (0.27-4.59)		0.862				
DM														
Yes		66		24 (36.36)		42 (63.64)		1						
No		79		40 (50.63)		39 (49.37)		1.39 (0.94-2.04)		0.09		0.97 (0.82-1.16)		0.79
BM (kg/m^2^)														
Underweight		65		39 (60.00)		26 (40.00)		2.1 (0.89-4.92)		0.08		1.29 (1.02-1.64)		0.03
Normal		56		21 (37.50)		35 (62.50)		1.31 (0.53-3.21)		0.55		1.07 (0.84-1.36)		0.57
Overweight		10		0		10 (100)						0.81 (0.64-1.03)		0.09
Obesity		14		4 (28.57)		10 (71.43)		1						
MUAC														
Normal		78		31 (39.74)		47 (60.26)		1						
Deficient		67		33 (49.25)		34 (50.75)		0.80 (0.55-1.16)		0.25		0.90 (0.77- 1.05)		0.20
Iron (mcg)														
Normal		15		4 (26.67)		11 (73.33)		1						
Deficient		130		60 (46.15)		70 (53.85)		1.73 (0.73-4.08)		0.21		1.26 (1.05-1.51)		0.01

## Discussion

The prevalence of anemia at the start of the ATT was 63.1% among our study participants, which differed from the finding of other studies conducted in Asia (prevalence ranging from 71.8% to 93.6%) [7,30-33], Africa (prevalence ranging from 62.2% to 71%) [8,17,18,34], and the Brazilian population (37%-89%) [5,23]. It is worth noting that while anemia is a common complication in PTB patients, differences in prevalence across populations may be attributed to factors such as environment, age, gender, socio-economic status, dietary practices, presence of chronic disease, bone marrow cellularity, as well as study design and sample size.

At the start of the ATT, the median (IQR) haemoglobin level among study participants was 11.9 (10.5-13.4) g/dL, with 10.9 (9.8-11.8) g/dL in anemic individuals and 13.9 (13.3-14.6) g/dL in non-anemic participants. We observed a significant improvement in Hb levels by the end of the ATT overall from 11.9 (10.5-13.4) g/dL to 13 (11.4-14.6) g/dL, and among the anemics from 10.9 (9.8-11.8) g/dL to 11.1 (10.15-11.8) g/dL. By the end of the ATT, hemoglobin levels of more than 55% of participants had improved.

To our knowledge, studies related to change in hematological parameters with ATT are few. Consistent with our finding, a study conducted by Manjunath and Patwegar also showed significant improvement in hemoglobin levels at the end of the intensive phase from 10 (8.40-12.57) g/dL to 11.8 (10.8-12.9) g/dL [15]. A decrease in the inflammatory response due to ATT leads to the improvement in Hb levels. However, a better nutritious diet following improvement in appetite also plays a vital role in improving Hb levels. Contrary to this report, a study conducted by Kassa et al., in Ethiopia, showed a reduction in Hb levels after two months of ATT; the study reported that the mean Hb level at the start and end of the intensive phase was 12.7 (2.09) g/dL and 11.8 (1.68) g/dL, respectively. The decrement in Hb levels may be drug-induced as the ATT drug can cause a variety of hematological disorders affecting RBCs [12].

Our findings indicate a notable decrease in the prevalence of anemia among patients with pulmonary tuberculosis following anti-tuberculosis treatment. The percentage of participants with anemia decreased from 63% to 44%. Similar to this finding, a study by Lee et al. from Korea reported a much higher reduction of 65.6% after ATT without iron intake [16]. A study from Brazil also reported that more than half of the study participants remained anemic at the end of the ATT [17]. In our study, at the end of the ATT, only three (2%) study participants who were nonanemic at baseline developed anemia at the end of the ATT, whereas a study conducted by Fernando et al. reported a much higher percentage of non-anemic PTB patients (36%) who turned anemic at the end of the ATT.

Peripheral smear analysis of blood samples showed that NCNC was the most common type of anemia (63.96%). Many studies around the world also reported that NCNC was the commonest type of anemia among PTB patients [7,16,31,33-35]. We noted that macrocytic anemia was the least prevalent type of anemia (1.8%) in our cohort. In line with our results, studies conducted by Yaranal et al. (4.05%), Dileepan et al. (3.3%), and Thatoi and Khadanga (4%) too reported macrocytic anemia among PTB patients [14,31,35]. Results of our study suggest that anemia among the PTB patients at the start of the ATT might be mainly due to the chronic disease. Several potential causes of TB-associated anemia have been proposed, but studies have consistently shown that inflammatory mediators reduce erythropoiesis and alter iron metabolism, leading to mild to moderate anemia [36,37]. In addition to these factors, causes of anemia reported are worm infestation, nutritional deficiencies, and malabsorption syndromes that exacerbate iron deficiency anemia. Tullius et al. and Jones et al. reported that MTB can utilize heme as an iron source for its survival in the host, which may also contribute to hemoglobin reduction [38,39]. Decreased MCV levels and increased RDW may be indicative of nutritional deficiencies in iron, folate, and vitamin B12 [40]. In fact, low dietary intake and low Hb levels were observed specifically among female participants.

Dietary intake assessment is a crucial component of a pulmonary tuberculosis treatment plan, along with the evaluation of lifestyles and hemoglobin levels, yet it is overlooked or underutilized. The results of 24-hour dietary analysis revealed that a significant proportion of NSP PTB patients in our cohort were deficient in micro- and macronutrients. Within two weeks of starting the ATT, over 95% of NSP PTB patients were found to have an energy intake deficiency and 73% had a protein intake deficiency. Although there was a significant improvement in nutrient intake levels observed among study participants after the ATT, the levels did not reach adequacy. Even after the completion of the ATT, deficiencies in energy, iron, calcium, and vitamin C intake were still prevalent, with rates of 87%, 66%, 86.8%, and 59%, respectively. However, there was a notable improvement in protein intake, with 80% of PTB patients reaching adequate levels after completing the ATT. When categorizing anemia based on its morphological types, it was observed that deficiencies in energy, iron, vitamin B12, and folate were more prevalent among the MCHC group, at 96.1%, 88.4%, and 96.1%, respectively. A low intake of iron by the end of the ATT was strongly associated with negative hemoglobin levels. The appetites of participants had improved significantly by the end of the antitubercular treatment, but their dietary intake had not reached the RDA, which was reflected in the increasing iron deficiency anemia among participants at the end of the ATT. Using binary logistic regression analysis, we found that female sex, economic status of BPL, underweight and deficiency in iron intake were the significant predictors of negative hemoglobin levels. All these conditions are consistent with a lower nutritional intake, and therefore, support the finding of persistence of iron deficiency anemia at the end of the ATT with most anemia of inflammation cases having recovered with the ATT.

This study also had certain limitations. Guidelines for the RDA have changed in year 2017 for the general population in India. There was a loss of 17.6% (31/176) of the participants during the follow-up; also, there could have been a social desirability bias in reporting the dietary intake. However, all efforts were taken to build a good rapport with the patients before collecting the data so as to get the valid responses.

## Conclusions

Anemia was present among three-fourths of NSP study participants at the start of the ATT. Although anemia was resolved in 22% of the NSP PTB patients at the end of the ATT without iron folic supplementation, 45% had persistent anemia of which most had iron deficiency anemia. There is a need to include screening of anemia and identifying the types and treatment of anemia along with ATT because anemia can worsen disease outcomes and predispose the patients to adverse treatment outcomes. At the end of the ATT, study participants' food consumption had improved, but it still did not meet the RDA. Hence, dietary counselling along with ATT needs to be followed.
